# The implementation of MOSAIQ‐based image‐guided radiation therapy image matching within radiation therapy education

**DOI:** 10.1002/jmrs.434

**Published:** 2020-09-26

**Authors:** Crispen Chamunyonga, Peta Rutledge, Peter J. Caldwell, Julie Burbery

**Affiliations:** ^1^ School of Clinical Sciences Queensland University of Technology Brisbane Queensland Australia

**Keywords:** Radiation therapy, IGRT, education, MOSAIQ, planar 2D images, CBCT

## Abstract

Image‐guided radiation therapy (IGRT) technologies are routinely used by radiation therapists (RTs) in clinical departments. However, there is limited literature on the acquisition and assessment of IGRT image‐matching competencies in undergraduate educational environments. This commentary paper aims to share the authors’ experiences in the development of teaching IGRT and image‐matching concepts in an undergraduate radiation therapy programme. It outlines how MOSAIQ oncology information systems (OIS) have enabled the university to embed hands‐on IGRT image matching on a range of clinical cases. The hands‐on exposure to case‐based planar and volumetric kilovoltage (kV) image matching has resulted in improved teaching and better preparation of students for clinical IGRT encounters. Students are likely to benefit from critical image assessment and decision‐making as well as the improved engagement in teaching and learning.

## Graphical abstract

The aim of this commentary paper is to share the authors’ experiences in the optimisation of teaching image‐guided radiation therapy (IGRT) and image‐matching concepts in an undergraduate radiation therapy programme. It outlines how MOSAIQ oncology information systems (OIS) enabled the university to embed in undergraduate teaching, hands‐on IGRT image matching on a range of clinical cases. The hands‐on exposure to case‐based planar kilovoltage (kV) and cone‐beam computed tomography (CBCT) image matching and resulted in improved teaching and better preparation of students for clinical learning.

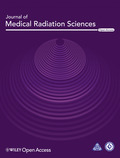

## Introduction

The role of image‐guided radiation therapy (IGRT) is well‐documented in the literature. It is through effective IGRT practices that random and systematic uncertainties are quantified and corrected to ensure accurate treatment delivery.^1^ The accuracy of image matching can be attributed to multiple factors including the quality of the images, the interuser variation in the application of image matching tools and use of automatic matching algorithms.^2^, ^3^, ^4^ The presence of disease, changes in tumour or nodal shape due to shrinkage and oedema can make the matching and decision‐making process challenging for the radiation therapists (RTs).^5^ The literature also highlights how cone‐beam computed tomography (CBCT) matching can take variable amounts of time depending on the RT experience and familiarity with the IGRT software.^6^ It is without doubt the knowledge and skill of the RT plays a significant role in the accuracy of treatment, based on decisions made at the time of online image review. Therefore, it is important to engage and equip undergraduate radiation therapy students with critical thinking skills and clinical judgement to undertake image matching competently.^7^, ^8^, ^9^

In undergraduate education, IGRT image‐matching skills and abilities are often acquired and developed in clinical settings utilising authentic clinical technology. Facilitating hands‐on experience with IGRT technologies in a university setting provides RT students with the opportunity to transfer theoretical knowledge gained into clinical practice. This was achieved through the integration of clinical software such as the MOSAIQ Oncology Information Systems (OIS) (IMPAC Medical Systems, Sunnyvale, CA), which is used in many clinical departments to support IGRT decision‐making, as well as tracking and reporting daily shifts. To our knowledge, the implementation of case‐based teaching with hands‐on IGRT image matching utilising MOSAIQ in a university setting has not been reported in the literature.

Following the development of IGRT teaching image matching at undergraduate level at Queensland University of Technology (QUT), we report successful implementation that has resulted in better teaching and learning approaches of IGRT image‐matching concepts. This also improved student engagement and provided students with the ability to practise IGRT image matching in a safe learning environment.

## Background

The set‐up of MOSAIQ for teaching into the Bachelor of Radiation Therapy programme was well aligned to QUT’s strong emphasis on the development of authentic teaching and assessment. MOSAIQ was funded by the university and installed on thirty (30) computer workstations for teaching. This was a great opportunity for the lecturers to develop case‐based volumetric IGRT teaching modules. Several MOSAIQ‐based learning activities were developed, which now form part of the curriculum. In 2017, a major review of the image‐matching teaching tasks resulted in a decision to embed more hands‐on image‐matching tasks into teaching for 2018. To improve pre‐clinical IGRT teaching, a greater educational database of planar megavoltage (MV) and kilovoltage (kV) and kV‐CBCT images were acquired to enable MOSAIQ‐based teaching to be implemented over several semesters in the four‐year programme.

An important task was to undertake a review of the literature to learn from several studies that reported and discussed the rationale for image matching competency training in clinical environments. Several articles reviewed highlighted it is imperative RTs are trained in IGRT, particularly image analysis and decision‐making.^7^, ^8^, ^9^, ^10^, ^11^, ^12^ An enquiry into the clinical use of IGRT technology and MOSAIQ OIS was important as we needed to understand how we would embed MOSAIQ and actual clinical practice through a range of teaching and learning strategies, whilst understanding the tools which would effectively support teaching. Using the MOSAIQ tools, we developed case‐based teaching modules for a range of patient scenarios. As shown in Table 1, the aim was to ensure students are exposed to a range of relevant and authentic learning opportunities with new modules added each year.

**Table 1:** Current MOSAIQ‐based planar kV and CBCT educational modules.

Anatomical Region	Planar kV modules	kV‐CBCT modules
Head and Neck	*Head and Neck*	*Head and neck with nodes (SIB)* *Brain*
Thoracic	*Lung* *Breast*	*Lung*
Abdomino‐pelvic	*Rectum* *Prostate* *Gynaecological* *Abdomen*	*Rectum with nodes* *Prostate* * *Gynaecological with nodes*
Other	*Spine (Thoracic and Lumbar)*	* *Thigh* * *Knee*

A*bbreviations:* CBCT: cone‐beam CT, kV: kilovoltage, SIB; simultaneous integrated boost.

* new modules.

### MOSAIQ tools and image matching

MOSAIQ has several image matching and visualisation tools for planar image matching, point, curve, greyscale and manual registration, with the ability to fuse the images and display the chequerboard, graticule and field apertures. Each semester, students are provided with comprehensive module handbooks, created by the university lecturers. These contain a step by step guide for the students to work through the planar and volumetric image‐matching tasks. Further information provided includes instructions for performing the image‐matching task, the image analysis tools to use and the matching techniques. Each student is allocated patients in MOSAIQ for them to analyse the reference digitally reconstructed radiographs (DRRs) and planar kV images, fuse the images, and record the shifts and comments in a spread‐sheet provided. They comment on whether the match is within tolerance, a move is required or whether the images need re‐acquiring. Students perform image matching of single images and orthogonal image pairs using MOSAIQ tools for 2D matching.

For three‐dimensional (3D) image matching, each student will access the allocated patients for image matching and refer to their workbooks for specific instructions on automatic and manual matching. These also stipulate whether a soft tissue or bony match is required. Using the MOSAIQ’s image‐matching tools and viewing options such as colour blend, quartered, chequerboard and spyglass, they align the planning computed tomography (CT) and CBCT datasets and make a clinical judgement regarding accuracy of the image registration. Similar to the requirements for 2D matching, students record their results and document their decisions. Results documented include translational and rotational shifts which include pitch, yaw and roll and any anatomical changes. The students also learn how to use the clip box tool in MOSAIQ to apply automatic registration to a selected region of interest using either a maximising mutual information or chamfer algorithm. In this way, teaching provides an understanding of the correct use of the tools and the limitations of relying on automatic matching tools in clinical decision‐making scenarios.

## Implementation Process

The university clinical partners were part of this process and provided the support required to achieve the outcome. Expertise from the clinical departments included RTs who ensured appropriate cases were selected for teaching. Information technology staff from the hospital and university, as well as vendor support, ensured the correct MOSAIQ set‐up and export/import of digital imaging and communications in medicine (DICOM) datasets. The process involved appropriate anonymisation and duplication of parent datasets consisting of reference CT data, plan dose and RT structure sets as well as the planar kV and kV‐CBCTs and generation of a unique instance identifier (UID) attribute for all datasets. The destination DICOM device settings, such as the local application entity (AE), the port and localhost settings, had to be checked for successful export/import. Patient registration and MOSAIQ data import were similar to the clinical settings where, prior to exporting a treatment plan from the planning software to MOSAIQ, the patient’s demographic data must be registered in MOSAIQ ensuring the attributes match the data with a registered patient name in MOSAIQ. Hence, before exporting CBCT data into MOSAIQ, the patients in MOSAIQ had to match the demographic data of the CBCTs. Once the images were imported into MOSAIQ, treatment fields and a site set‐up definition was generated and approved mimicking an authentic clinical workflow.

## Case‐based Learning

Several case‐based image‐matching modules with hands‐on teaching were developed which incorporated planar (kV and MV) and volumetric (kV‐CBCT) image‐matching tasks. Students are allocated a range of patients in MOSAIQ to perform matching tasks individually, ensuring that all students have the opportunity to participate ‘hands‐on’. We found the best solution was to provide specific details such as the set‐up and immobilisation, diagnosis, care plan and dose prescription for each module. This is consistent with the case‐based approach recommended in the literature^9^, ^13^, ^14^ and the National Radiotherapy Implementation Group (NRIG) recommendations.^9^

It was beneficial that students access a range of daily CBCTs for each patient case. For example, a head and neck case was set‐up so students could access 33 days of daily CBCTs. This made it possible for the students to assess geometry and anatomic changes, challenging the students to re‐think the impact of weight loss, tumour shrinkage or other changes that can cause a discrepancy between the CBCT and the pre‐treatment CT.^15^ Similarly, in pelvic patients, students can assess changes to the patient and comment on compliance with a bladder filling and rectum emptying protocol. During the development of the image‐matching modules, two RT lecturers assessed the images independently to generate ‘expert matches’ which are now used as a baseline to assess the level of accuracy achieved by the students. The use of MOSAIQ in teaching made it easier to strengthen teaching on decision‐making for a range of clinical cases incorporated into teaching and embed image matching as part of the students' assessment. As higher education literature highlights, feedback and assessment are paramount to learning.^16^, ^17^, ^18^ During the face‐to‐face image‐matching sessions, students receive timely feedback and guidance from the lecturers. Assessment of IGRT competency was incorporated as recommended by the NRIG training programme framework.^9^ It is expected that this will further encourage the students to engage in learning.^17^ Online quizzes were developed to assess students’ understanding of the IGRT workflows and decision‐making process. The implementation of MOSAIQ also enabled the students to perform image matching in self‐directed sessions with protected time allocated to access the computer laboratory. Students’ participation in self‐directed activities has been encouraging, and anecdotal feedback provided by the students has been positive.

**Figure 1:**
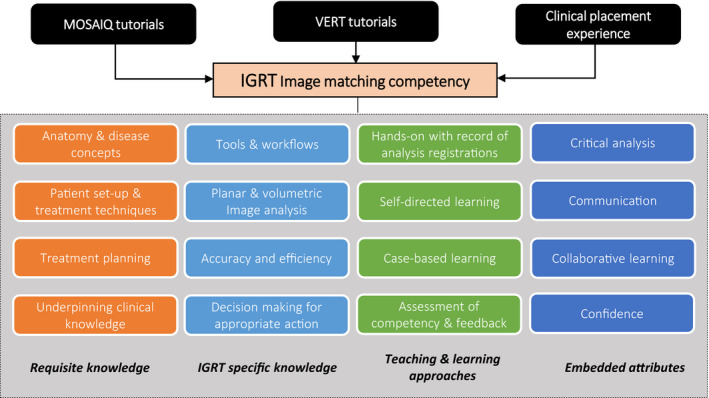
Preliminary institutional framework for IGRT image‐matching competence development in undergraduate radiation therapy education.

There was a need to provide didactic content specific to IGRT and align teaching to other units within the programme. This also involved an analysis of the contributions Virtual Environment for Radiotherapy Training (VERT) makes in the teaching of image matching concepts. Figure 1 shows several other important elements that were considered necessary for effective teaching. These include requisite knowledge such as anatomy and treatment planning necessary for obtaining an accurate match and decision‐making. Other elements include efficient use of image matching tools, critical analysis, improving communication and collaboration and confidence.^19^ For this to occur, a range of teaching and learning approaches were necessary and were informed by higher education literature.^16^, ^17^, ^18^ This also ensured that students are engaged, motivated and that learning is scaffolded. Further work is being undertaken to gather empirical evidence on students' competency and experiences in the MOSAIQ‐based image‐matching activities to inform this preliminary framework.

## Summary

With support from the clinical partners, the university was able to develop more authentic and engaging IGRT image‐matching tasks for the students. To further improve teaching and learning, we are extending the range of case‐based planar and volumetric image‐matching teaching to a variety of tumour sites, incorporating new cases to ensure clinical relevance. Continued emphasis on strengthening teaching and learning strategies and building the students’ confidence is important. However, the main challenge in the implementation process is the time‐consuming task of manually creating pseudo‐patients and the duplication of the kV and CBCT images sets, to allow each student to have their own case and set of images to practise image matching. Despite this, creating these image matching activities was necessary to ensure are well‐prepared for clinical image‐matching tasks. The lecturers acknowledge this pre‐clinical case‐based teaching in university environments does not replace real‐world online clinical scenarios; however, it results in better preparation before clinical placement.

## Conclusions

The changes in radiation therapy technology and clinical protocols necessitate equipping radiation therapy students with knowledge and skills in IGRT decision‐making to ensure safe and accurate delivery of radiation therapy. Embracing the challenges of handling clinical software in university environments, we were able to embed hands‐on image matching that provided more effective and comprehensive teaching. As the literature also suggests, more emphasis needs to be placed on the development of more robust IGRT teaching, incorporating image analysis, decision‐making and relevant theory that underpins knowledge of image matching and IGRT. This may be achieved through the establishment of increased collaborative partnerships with clinical departments to share ideas, resources and develop tools necessary to enhance the quality of teaching and learning and improve skills critical to success in students’ future clinical roles.

## Funding Information

This review received no grant from any funding agency, commercial or not‐for‐profit sectors.

## Conflicts of Interest

The authors declare no conflict of interest.
